# Editorial: Actinobacteria in Special and Extreme Habitats: Diversity, Function Roles and Environmental Adaptations, Second Edition

**DOI:** 10.3389/fmicb.2019.00944

**Published:** 2019-04-30

**Authors:** Sheng Qin, Wen-Jun Li, Hans-Peter Klenk, Wael N. Hozzein, Iftikhar Ahmed

**Affiliations:** ^1^The Key Laboratory of Biotechnology for Medicinal Plant of Jiangsu Province, Jiangsu Normal University, Xuzhou, China; ^2^State Key Laboratory of Biocontrol and Guangdong Provincial Key Laboratory of Plant Resources, School of Life Sciences, Sun Yat-sen University, Guangzhou, China; ^3^Southern Laboratory of Ocean Science and Engineering (Guangdong, Zhuhai), Zhuhai, China; ^4^School of Natural and Environmental Sciences, Newcastle University, Newcastle upon Tyne, United Kingdom; ^5^Bioproducts Research Chair, Zoology Department, College of Science, King Saud University, Riyadh, Saudi Arabia; ^6^Botany and Microbiology Department, Faculty of Science, Beni-Suef University, Beni-Suef, Egypt; ^7^National Agricultural Research Center, Bio-resources Conservation Institute, Islamabad, Pakistan

**Keywords:** actinobacteria, special and extreme environments, diversity, omics technologies, phylogenomics, activities, environmental adaptation

*Actinobacteria* produce structurally diverse bioactive natural products, such as enzymes, antibiotics, antitumor and immune regulatory agents. *Actinobacteria* are not only the main producers of microbial-derived drugs, they also play an important role as symbionts in plant-associated microbial communities (Barka et al., 2015). At the same time, members of the phylum *Actinobacteria* were found to be widely distributed in different ecological environments, including diverse special and extreme habitats of aquatic and terrestrial ecosystem (Qin et al., 2011; Dhakal et al., 2017; Goodfellow et al., 2018). Compared with actinobacteria from temperate habitats, the community structure, diversity, biological activities, and mechanisms of environmental adaptation of those actinobacteria in special and extreme environments are relatively unstudied and unclear, and their functions and utilization are even less reported. These actinobacteria are potential new sources of novel natural products and functions for exploitation in medicine, agriculture, and industry.

It's exciting that there are more and more reports in this field recently. These discoveries make us consider some intriguing and new questions, such as, are actinobacteria ubiquitous in the special and extreme environment on Earth, and where are the limits of their survival? At the same time, the discovery of more and more pure cultures and new taxa of actinobacteria from extreme environments has raised new questions for the taxonomy of the phylum *Actinobacteria*. How can we establish a more accurate taxonomic system to reflect the natural evolutionary relationship of *Actinobacteria*? Moreover, how can we recognize the specific ecological functions of these ecologically adapted actinobacteria and their potential unique environmental adaptation mechanisms? Following the success of the Research Topic, “Actinobacteria in special and extreme habitats: diversity, functional roles, and environmental adaptations” (Qin et al., 2016), organized in 2015, we are happy to launch a second edition. More than 100 authors, from 14 different countries, contributed a total of 16 articles in this new edition, including one review paper and 15 original research articles, covering a variety of topics related to actinobacteria in special and extreme habitats. These articles addressed issues related to the cultivation methods of rare actinobacteria, metagenomic analyses of diversity, phylogenomic taxonomy, genome mining, bioactive compounds, and their habitat adaptation mechanism using omics approaches. We are grateful to all authors who have submitted their manuscripts to the second edition of this Research Topic.

The special and extreme environments are likely to contain abundant rare actinobacteria and novel species. However, the acquisition of pure culture is a prerequisite for the further study of their classification and function. Caves spread all over the world, being dark, humid, and nutrient-limited. The cultivation of these cave microorganisms has proven to be challenging (Ghosh et al., 2017). An original article by Fang et al. explores the effects of heat pretreatment, pH, and calcium salts on isolation of rare actinobacteria from Karstic Caves in Yunnan, China. A total of 204 isolates were cultured, and the authors obtained a high number of 29 different rare actinobacterial genera. Actinobacteria from caves have been found to produce a variety of secondary metabolites. However, studies of microbial ecology in caves are still very limited. Recently, members of actinobacteria were reported to be possibly involved in the moonmilk genesis (Bindschedler et al., 2014). Interestingly, the article by Maciejewska et al. provides novel evidences that some filamentous *Streptomyces* could be key protagonists in the genesis of moonmilk through a wide spectrum of biomineralization processes. These studies enlarged our knowledge on cave actinobacterial diversity and their special ecological functions. Desert is the most extreme non-polar biome on Earth. Recent metagenomic analyses of hyper-arid and extreme hyper-arid desert soils revealed a remarkable degree of actinobacterial “dark matter” (Idris et al., 2017). The diversity of actinobacterial taxa in the Badain Jaran (BJD) and Tengger Deserts (TGD) of China were assessed using combined cultivation-dependent and high-throughput sequencing techniques (Sun et al.). These authors found that the phylum *Actinobacteria* was the predominant, comprising 35.0 and 29.4% of the communities in the two desert sands, respectively. Taxonomic classification of 1,162 actinobacterial strains revealed a high diversity of 73 genera, including 37 new taxa, and 10.36 % of the tested isolates showed antimicrobial activities (Sun et al.). However, their ecological significance in deserts deserved further exploration.

Marine actinobacteria have attracted more and more attention because of their special physiological characteristics and capacity of producing various natural compounds with diverse bioactivities (Schinke et al., 2017). However, marine actinobacteria producing anti-complement agents are still poorly explored. Xu et al. analyzed the genome of a marine *Streptomyces* sp. DUT11, which showed a strong anti-complement activity, and isolated the active compounds tunicamycins I, V, and VII. Another marine actinobacterium, *Glycomyces sediminimaris* UTMC 2460, which showed anti-microfouling activity, was analyzed for its active compounds. These authors concluded that diketopiperazines produced by this strain could be used as environmentally safe anti-fouling agents to prevent the fouling process in marine habitats (Heidarian et al.). The article by Sun et al. reveals the marine adaptation mechanism of a sponge-derived actinobacterium, *Kocuria flava* S43, by comparative genomics analysis. These authors found that gene acquisition was probably a primary mechanism of environmental adaptation in *K. flava* S43 (Sun et al.). These studies indicated that marine actinobacteria are rich sources of diverse biological compounds.

In this Research Topic, we collected five papers related to endophytic actinobacteria, which is also a research hotspot in recent years. Habitat-adapted, symbiotic, indigenous endophytic actinobacteria from special and extreme habitats probably contain novel taxa and compounds, and enhance their host tolerance of harsh environments (Mesa et al., 2017; Qin et al., 2018). The article by Singh and Dubey reviews the taxonomic and chemical diversity of endophytic actinobacteria in arid, mangrove, non-mangrove saline and aquatic ecosystems and discusses their potential biotechnological applications. Similarly, Jiang et al. explores the diversity and antibacterial activities of endophytic actinobacteria from five different mangrove plants in Guangxi Zhuang Autonomous Region, China; they found 28 actinobacterial genera and four potential new species. The two articles by Bibi et al. and Wei et al. report on the endophytic actinobacteria and their biological secondary metabolites from the halophyte *Salsola imbricate* and Chinese tea plants; their results confirm again that endophytic actinobacteria might be an undeveloped bioresource library for active compounds. Lasudee et al. report the actinobacteria associated with arbuscular mycorrhizal spores of *Funneliformis mosseae*, and explore their potential plant growth promotion effects in agriculture; results showed that the isolates could produce indole-3-acetic acid (IAA) and siderophores, solubilize phosphate, and promote rice plant growth.

Genome sequencing and the phylogenomic strategy have been explored for the research of taxonomy and prokaryotic systematics. For instance, the class *Acidimicrobiia* is comprised of few cultivable species at present, containing only the order *Acidimicrobiales*, two families *Acidimicrobiaceae* and *Iamiaceae* with few genera (Ludwig et al., 2012). Hu et al. analyzed 20 sequenced members of this class and identified 15 conserved signature indels (CSIs) in widely distributed proteins and 26 conserved signature proteins (CSPs); the phylogenomic analysis revealed another three major lineages in addition to the two recognized families. Furthermore, Sangal et al. revisit the taxonomic status of the biomedically and industrially important genus *Amycolatopsis*, using a phylogenomic approach. According to the genome sequences analysis and the core genome phylogeny, genus *Amycolatopsis* was subdivided into four major clades and several singletons (Sangal et al.). These results indicate that whole genome sequencing analysis can provide more accurate taxonomic status for prokaryotes.

The developments of omics methods have provided a robust support for our understanding of the actinobacterial adaptation mechanisms to the special and extreme habitats. Cornell et al. obtained 76 plasmid-containing isolates of actinomycetes from the Great Salt Plains of Oklahoma. Eleven isolates were chosen for genome sequencing, and the results revealed the presence of series genes involved in antibiotic production, antibiotic, and heavy metal resistance, osmoregulation, and stress response, which likely facilitate their survival in the extreme halophilic environment (Cornell et al.). By transcriptome analysis, physiological, and molecular experiments, Han et al. found that accumulation of ectoine played a vital role for the salt stress tolerance of the halotolerant *Nocardiopsis gilva* YIM 90087^T^. The article by Yin et al. report that a hybrid strategy was used to utilize carbon sources at different temperatures by an aerobic, and cellulose degrading thermophilic actinomycete, *Thermoactinospora rubra* YIM 77501^T^, by using combined genomic and transcriptomics methods.

In summary, this Research Topic second edition presents recent discoveries on diversity, function roles, and environmental adaptations of actinobacteria in special and extreme habitats; and broadens our knowledge of actinobacterial diversity and their ecophysiological function. We are delighted to present this Research Topic in Frontiers in Microbiology. We hope that readers of the Journal will not only enjoy this Research Topic but also will find it a useful reference. Future research still looks forward to the innovation and application of new technologies, such as the application of single cell microfluidic technique to obtain new pure cultures. At the same time, the cooperation of different disciplines, and international cooperation of scientists from different countries should be strengthened. We also believe that in the future, more “dark matter” from actinobacteria in special and extreme environments will be discovered and utilized for the benefit of human beings.

## Author Contributions

W-JL and SQ organized this topic. SQ wrote the editorial article. H-PK, WH, and IA are co-editors of the topic and discussed the writing.

### Conflict of Interest Statement

The authors declare that the research was conducted in the absence of any commercial or financial relationships that could be construed as a potential conflict of interest.
